# Effects of STN DBS and auditory cueing on the performance of sequential movements and the occurrence of action tremor in Parkinson’s disease

**DOI:** 10.1186/1743-0003-11-135

**Published:** 2014-09-11

**Authors:** Tjitske Heida, Eva Christine Wentink, Yan Zhao, Enrico Marani

**Affiliations:** MIRA Institute for Biomedical Engineering and Technical Medicine, University of Twente, Department of Electrical Engineering, Mathematics and Computer Science, Biomedical Signals and Systems group, P.O. Box 17, Enschede, Netherlands

**Keywords:** Parkinson’s disease, Action tremor, Power spectral density, Auditory cueing, Deep brain stimulation, Subthalamic nucleus

## Abstract

**Background:**

Parkinson’s disease (PD) patients show a higher ability to perform repetitive movements when they are cued by external stimuli, suggesting that rhythmic synchronization with an auditory timekeeper can be achieved in the absence of intact basal ganglia function. Deep brain stimulation (DBS) is another therapeutic method that improves movement performance in PD and may suppress or enhance action tremor. However, the combined effect of these therapies on action tremor has not been studied yet. In this pilot study, we thus test the effect of both DBS in the subthalamic nucleus (STN) and auditory cueing on movement performance and action tremor.

**Methods:**

7 PD patients treated with (bilateral) STN DBS were asked to move one hand or foot between two dots, separated by 30 cm as indicated on the table or the floor. The movement frequency was dictated by a metronome with a frequency in the range of 1.6 to 4.8 Hz. Each test was repeated three times for each extremity, with different stimulation settings applied during each repetition. The power spectral density patterns of recorded movements were studied. Tremor intermittency was taken into account by classifying each 2-second window of the recorded angular velocity signals as a tremor or non-tremor window. By determining the phase locking value it was tested whether movement or tremor was synchronized with the auditory cue.

**Results:**

While action tremor presence or absence did not affect the level of synchronization of the movement signal with the auditory cue for the different metronome frequencies, the number of extremities showing action tremor was significantly reduced under external cueing conditions in combination with DBS. In this respect the cueing frequencies of 1.6 and 4.8 Hz showed similar effects, suggesting that the frequency of the cueing signal is not that critical.

**Conclusion:**

The combination of deep brain stimulation and auditory cueing, which both are proposed to involve the activation of cerebellar circuits, shows an enhanced action tremor reduction in Parkinson’s disease.

**Electronic supplementary material:**

The online version of this article (doi:10.1186/1743-0003-11-135) contains supplementary material, which is available to authorized users.

## Introduction

The basal ganglia play an important role in the sequencing of repetitive motor tasks [1, 2]. For well-learnt predictable sequences it has been suggested that the basal ganglia provide an internal non-specific cue to switch between consecutive movements in a movement sequence, and to develop preparatory activity for each sub-movement in the sequence [3]. In Parkinson’s disease (PD), the function of the basal ganglia is affected and thus this internal rhythm formation is often disturbed. As a result, PD patients frequently have problems initiating and maintaining a steady movement rhythm on their own. For instance, many PD patients suffer from locomotor deficits like freezing of gait and gait festination [1, 4, 5]. Bradykinesia and akinesia may also be partly attributed to this defective internal cueing which disrupts and impairs the outflow of motor responses [4, 6, 7].

It is known that PD patients may benefit from external cues in the form of temporal or spatial stimuli, initiating movements and pacing repetitive motor sequences [1, 5, 8–12]. In fact, PD patients experiencing bradykinesia start to rely more on visual guidance for their movements [6, 13]. For example, parallel lines on the floor perpendicular to the walking direction have been shown to greatly increase gait velocity and stride length [14, 15]. Similarly, patients who synchronize their steps to the beat of a metronome show improved quality of walking, i.e. increased velocity and stride length and decreased cadence [16] and less freezing episodes [17]. That PD patients can better perform repetitive movements in the presence of auditory or visual stimuli suggests that rhythmic synchronization with external cues can be achieved in the absence of intact basal ganglia function [5, 8, 18].

Deep brain stimulation (DBS) is another therapeutic method that may improve movement performance in PD patients. DBS in the subthalamic nucleus (STN) or globus pallidus internus (GPi) are currently accepted treatments for medically intractable PD, reducing PD symptoms and improving motor complications accompanying (long-term) levodopa treatment [19–22]. Although its exact mechanism of action is not known, it has been proposed that stimulation-induced modulation of pathological network activity underlies the therapeutic effect of DBS [23–26].

In a few studies the combination of DBS and cueing therapy on movement performance have been tested, in part to investigate if these types of therapies address different neuronal circuits. Nowak et al. have investigated the combined effect of cueing and STN DBS on the performance of arm and leg movements in PD patients (e.g. grasping to lift an object) [27]. They found that this combination of therapies increased the speed of grip force development as well as the acceleration of the lifting movement. Specifically, akinesia of grasping movements was improved by auditory cues for both DBS on and off. Based on these results, and in accordance with previous studies, it was suggested that the auditory cues act as, or activate a compensation mechanism that bypasses the defective basal ganglia. Schenk et al. showed similar results by comparing the effect of GPi DBS on internally versus externally timed movements [28]. In their study reach and grasp movements were performed with stationary and moving target objects. Reaching and grasping of stationary objects was suggested to rely on internal timing, while external timing signals were provided by the moving targets. Movement performance improved when external cues were provided with the effect of DBS less pronounced than with internal timing. Using the effectiveness of GPi DBS as an indicator of the involvement of the basal ganglia, it was suggested that the improved motor performance demonstrated that the basal ganglia were less involved in the control of externally timed movements [28].

While movement may generally suppress rest tremor [29–31], PD patients may also suffer from action tremor, tremor that occurs during voluntary movements. Recently, we used movement recordings from the hands and feet at rest and during a simple tapping movement to show that DBS may suppress or alternatively enhance rest and/or action tremor. This is reflected in a shift in the movement power from the tremor frequency band (3.5-7.5 Hz) to the low frequency band (<3.5 Hz) during tremor suppression or vice versa during tremor enhancement [32]. Likewise, this shift in movement power also occurred for rest tremor, for which it may have been expected as found in other studies, that effective DBS may restore normal physiological tremor, and thus resulting in a shift of power toward frequencies within the frequency band of 7.5-15 Hz, and decreased tremor regularity [19, 33–35].

The combined effect of DBS and cueing on action tremor has not been studied yet. The aim of this pilot study was therefore to investigate the effect of auditory cueing on the performance of repetitive arm and leg movements and the occurrence of action tremor in the same limb in a group of PD patients treated with (bilateral) STN DBS. The frequency of the auditory cues ranged from those found during normal movements to the parkinsonian tremor frequency. Clinically effective and less or non-effective settings of DBS were applied.

## Methods

### Subjects

A total of 7 PD patients participated in this study, with an average age of 63 ± 6.5 years. At least three months prior to the experimental measurements, patients underwent DBS lead (Medtronic 3389) implantation in the STN. All but one patient received bilateral DBS. All patients satisfied the following criteria:

-  Positive and fast (within 5 min.) response to DBS;
-  No major fluctuations in their motor symptoms due to medication;
-  In fit physical condition and able to fully cooperate during the experiments;
-  No dementia and/or dyskinesia diagnosed during DBS treatment.

Medications were not withheld before the measurement session. All procedures conformed to the Declaration of Helsinki and were approved by the Medical Ethical Committee of the Medisch Spectrum Twente in Enschede, the Netherlands. All subjects gave informed consent in advance. For more patient details see Tables 1 and 2.

**Table 1:** **Patient details (time in years)**

Pat.	Sex	Age	Disease dur.	Time after surg.	Targ.	DBSon	DBS80%	DBSoff
1	F	68	15	6	R	2.0 V, 60 μs, 140 Hz, 4-C+	1.6 V	off
2	M	62	16	6	R	3.6 V, 60 μs, 140 Hz, 1-C+	2.9 V	off
					L	3.9 V, 60 μs, 140 Hz, 5-C+	3.1 V	off
3	M	61	17	1	R	3.0 V, 60 μs, 145 Hz, 1-C+	2.4 V	off
					L	2.8 V, 60 μs, 145 Hz, 1-2-C+	2.2 V	off
4	F	62	6	3	R	2.5 V, 60 μs, 145 Hz, 1-C+	2.0 V	off
					L	3.2 V, 60 μs, 145 Hz, 1-2-C+	2.6 V	off
5	F	75	13	1	R	3.5 V, 120 μs, 145 Hz, 1–2 + 3-	2.8 V	off
					L	3.3 V, 120 μs, 145 Hz, 1–2 + 3-	2.6 V	off
6	M	62	12	7	R	4.2 V, 90 μs, 140 Hz, 7-C+	3.4 V	off
					L	3.6 V, 60 μs, 140 Hz, 0-1-2-C+	2.9 V	off
7	M	54	18	6	R	3.4 V, 60 μs, 140 Hz, 1-2-C+	2.7 V	x
					L	4.0 V, 90 μs, 140 Hz, 6-7-C+	3.3 V	x

F: female; M: Male; R: right STN; L: left STN; C: stimulator case; x: not included in the experiment. Stimulation sites are indicated by 0, 1, 2, 3 corresponding to the four electrode contacts of the DBS lead on the left side, and 4, 5, 6, 7 on the right side in case of a single stimulator. Stimulation sites are indicated by 0, 1, 2, 3 for both sides in case separate stimulators for the left and right STN were used.

**Table 2:** **UPDRS scores**

Pat.	DBS setting	UPDRS				
		20	21	24	25	26
1	DBSon	0	1	x	x	x
	DBS80%	0	1	x	x	x
	DBSoff	0	1	x	x	x
2	DBSon	0	3	2	3	3
	DBS80%	0	3	2	2	2
	DBSoff	0	3	2	2	3
3	DBSon	0	0	1	1	1
	DBS80%	0	1	2	1	1
	DBSoff	0	0	2	1	1
4	DBSon	x	2	2	x	x
	DBS80%	2	2	2	2	4
	DBSoff	4	4	2	3	4
5	DBSon	0	0	3	2	2
	DBS80%	0	0	2	1	x
	DBSoff	0	0	2	2	2
6	DBSon	2	1	2	1	1
	DBS80%	3	1	2	2	2
	DBSoff	4	1	3	2	2
7	DBSon	0	0	3	3	3
	DBS80%	0	1	2	3	4
	DBSoff	0	x	x	x	2

20: rest tremor upper extremities; 21: action tremor upper extremities;

24, 25: hand movements; 26: foot movements.

### Data acquisition

Four inertial sensors (MT9^®^, Xsens Technologies BV, Enschede, the Netherlands) for measuring angular velocity were taped to the hands and feet of the patients and connected to the Xbus master (MT9^®^) placed around the waist; data was sent to a laptop via Bluetooth. All signals were filtered by a 20 Hz pre-sampling filter and sampled at 50 Hz.

### Auditory cueing test

Each patient was asked to move his or her hand or foot between two dots, separated by 30 cm as indicated on the table or floor. This test was sequentially performed by the right arm, left arm, right leg, and left leg. The movement frequency was dictated by a metronome implemented in Matlab (MathWorks, Inc., 2010) that beated at a frequency of 1.6, 3.2 or 4.8 Hz, in random sequential order, for 10 to16 seconds. Thus, the frequency of the auditory cues ranged between 1.6 Hz, which is within the range of frequencies found during normal movements like gait [36, 37] with an upper limit of around 3.2 Hz for healthy subjects [36], and 4.8 Hz, which is within the range of PD tremor frequencies. Each test was repeated three times, with different DBS settings each time:with n indicating the number of extremities tested for this setting. The order of the tests (i.e. the sequence of right/left arm, right/left leg) was randomized for each DBS setting while the order of the settings was randomized for each patient. These tests were part of a more extended measurement protocol, which also included a rest tremor test and a self-paced tapping test performed with the same DBS settings [32]. The total duration of all tests at a single setting was about 15 min. In between settings, patients were allowed to rest for about 5 minutes and adjust to the new DBS setting.

DBSon, Settings normally used by the patient (n = 26);

DBS80%, Stimulation amplitude reduced to 80% (n = 26);

DBSoff, Stimulator off (n = 22);

### Data analysis

All analyses were performed in Matlab (MathWorks, Inc., 2010). Prior to the analyses, all recordings were high-pass filtered with a cut off frequency of 0.25 Hz with a 2nd order non-causal Butterworth filter.

#### Classification of tremor and non-tremor windows

Although tremor often occurs intermittently [32, 38, 39], tremor frequency is rather constant [33, 35]. Therefore, tremor may be recognized within short time intervals based on the power spectral density (PSD) of the recorded movement signals. The results of a previous study have shown that tremor and non-tremor intervals are associated with distinct patterns in the power distributions of the recorded movement signals [32]. We therefore consider the presence and absence of tremor as two distinct conditions. All signals were divided into 2 second windows and each window was classified as a tremor or non-tremor window using an algorithm based on the method adapted from [38] and described in [32].

For each 2-second window the PSD was estimated using an all-pole 6th degree autoregressive model using the Burg method. The AR model enables the detection of resonance peaks that express the oscillatory behavior of a system. Therefore, classification of the tremor windows is based solely on the dominancy of the oscillatory behavior as a ‘system’ property of the extremity irrespective of the amplitude. Windows were classified as tremor windows when the dominant pole of one of the three axes of rotation exceeded a threshold of 0.88. The tremor frequency band and the threshold were selected based on the visual inspection of all 2-second windows of all patients. A threshold of 0.88 accommodates variations in the tremor frequency and amplitude as normally observed in PD patients.

In addition, the PSD (periodogram) was calculated for each window (using a Hann window) over a frequency range up to 15 Hz. The average PSD of the windows was calculated separately for tremor and non-tremor windows.

#### Power distribution

The power distribution of the angular velocity measurements were determined for three subdivisions in the 0–15 Hz frequency band as follows [32]:

-   < 3.5 Hz, the low frequency (LF) band, associated with voluntary movements [36];
-  3.5-7.5 Hz, the tremor frequency (TF) band, associated with rest and action tremor;
-  7.5-15 Hz, a high frequency (HF) band, associated with normal physiological tremor.

For each 2-second window of the angular velocity signal, the absolute and the relative power was calculated. The relative power in each of these frequency bands was calculated by dividing the absolute power in the respective frequency sub-band by the total power in the window over 0 to 15 Hz [32].

For tremor windows, the average tremor frequency was determined by averaging the peak frequencies found from the dominant pole while for non-tremor windows the mean frequency in the TF band was calculated. Moreover, the mean frequency for the LF and HF bands was calculated for both tremor and non-tremor windows. These parameters were determined for each of the four extremities for each test and DBS setting.

#### Phase locking value

Using the angular velocity measurements from the inertial sensors, it was determined whether the movements of the hands and feet of the patients were synchronized with the beat of the metronome. For two simultaneous signals to exhibit synchronization, a common cyclic pattern must be present in both signals, albeit with different phases. Thus, the phase locking value (PLV), the phase difference between two signals over short periods of time, was used as a measure of movement performance [40]. The metronome data was represented by a sinusoidal signal with the correspondinSg frequency. From the gyroscope data, the axis of rotation with the maximum amplitude was taken to represent the movement of the hand or foot. The phase locking value was calculated for epochs with a duration equal to one period of the sinusoid. According to the frequency bands defined described above, the recorded angular velocity signal was divided into a LF component using a low pass filter with a cut off frequency of 3.5 Hz (order 4 non-causal Butterworth) and a TF component using a 3.5-7.5 Hz band pass filter (order 4 non-causal Butterworth). Using the Hilbert transform, the instantaneous phase was determined for the gyroscope signal (*φ*_*gyr*_), either the LF or TF component, and the sinusoidal metronome signal (*φ*_*sin*_). The phase difference between either components of the angular velocity and the metronome signal was calculated according to

1 $$\Delta \varphi =\varphi_{\text{sin}}-\varphi_{\text{gyr}}$$

For each epoch, the phase locking value (PLV) was thus calculated according to

2 $$\text{PLV}\left(m\right)=\frac{1}{N}\left|\sum_{n=1}^{N}e^{-i\Delta \varphi_{n}}\right|$$

where *m* represents the epoch and *n* the samples within the epoch. The mean PLV was determined for the LF component of the angular velocity with a 1.6 Hz or 3.2 Hz cueing signal (*PLV*_*LF,1.6Hz*_ and *PLV*_*LF,3.2Hz*_, respectively) and the TF component with a 4.8 Hz cueing signal (*PLV*_*TF,4.8Hz*_).

**Figure 1:**
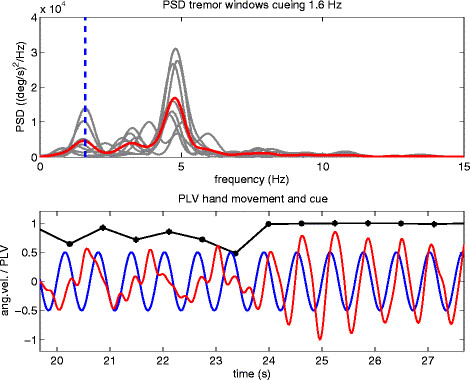
**Data recorded at the right hand of a patient while the stimulator was switched off (DBSoff).** The upper graph shows the PSDs of every 2 s-window of the recorded angular velocity (gray lines) and the average PSD of these windows (red line). All windows were in this case classified as tremor windows. The broken line indicates the frequency of the auditory cueing signal, i.e. 1.6 Hz. The lower graph shows the low frequency component of the angular velocity (red line), associated with voluntary movement performed by the hand. This movement component was extracted by filtering the angular velocity signal with a low pass filter (5th order non-causal Butterworth filter with a cut off frequency of 3.5 Hz) and normalizing the filtered signal to a range between -1 and 1. The cueing signal is represented by a 1.6 Hz sinusoidal signal (blue line) with an amplitude of 0.5. In addition, the PLV for the movement component of the angular velocity and the cueing signal (black markers connected by a linear line) is shown.

Figure 1 (lower graph) shows an example of such a PLV (black line with markers), representing the degree of synchrony between the hand movement of one of the patients and a 1.6 Hz auditory cue. Also shown are the sinusoidal cueing signal (blue line) and the normalized LF component (including frequencies <3.5 Hz) of the angular velocity recorded at the hand (red line) which is associated with voluntary movement. In instances where the PLV has a value near unity, the movement could be synchronized with the cueing signal.

Figure 1 (upper graph) shows the PSD of every 2-second window of the angular velocity over the total frequency range of 0–15 Hz (gray lines) and the average PSD of all these windows (red line). The 1.6 Hz cue is indicated in this graph by a blue broken line. In this particular case, all windows were classified as tremor windows. The peak at around 3.2 Hz corresponds to the higher harmonic of the movement signal.

### Statistics

For each cueing frequency and DBS setting, the average PLV and the relative power of the angular velocity was calculated for all four extremities of an individual patient and for each of the three frequency bands (i.e., low, tremor and high). Tremor and non-tremor windows were assessed separately. A Wilcoxon’s two-tailed rank-sum test with a significance level of 5% (p < 0.05) was used to compare the different conditions. This test was selected because the sample size varied for the different conditions and the data was not normally distributed for all cases. In some cases, the sample size was relatively low because not all patients showed tremor and non-tremor windows in each extremity. The Bonferroni correction was applied for multiple comparisons (n = 9). In the scatter plots, linear trend lines were determined using a robust fitting method [41]. The Chi-square test was used to compare the occurrence of tremor in any of the extremities for different cueing signals.

## Results

### Power distribution patterns

**Figure 2:**
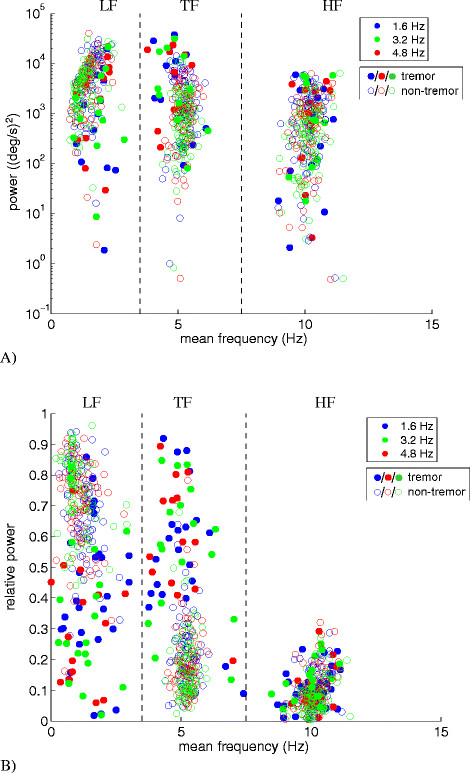
**The absolute (A) and relative (B) power of the angular velocity in 2 s windows as a function of the mean frequency in the 3 frequency sub-bands, delineated by dashed lines: the LF band (<3.5 Hz), the pathological tremor (TF) band (3.5-7.5 Hz), and the normal physiological tremor frequency (HF) band (7.5-15 Hz).** Each window was classified as a tremor (closed markers) or non-tremor (open markers) window. Tests were performed with auditory cues with different frequencies: 1.6 Hz (blue markers), 3.2 Hz (green markers), and 4.8 Hz (red markers). Each data marker represents a single extremity in individual patients at a particular DBS setting.

Figure 2 shows the absolute (Figure 2A) and relative (Figure 2B) power distribution of the angular velocity as a function of the mean frequency in the three specified frequency bands. Data for all metronome frequencies, all extremities and DBS settings are included in these graphs. Tremor (closed markers) and non-tremor (open markers) windows possess high power in the TF and LF bands, respectively: the relative power in the TF band was significantly higher in presence of tremor (p < 0.05), and was similar in both the hands and feet. These results are comparable to previous findings for tremor measured at rest (i.e. rest tremor) and tremor measured during self-paced hand or foot tapping (i.e. action tremor) [32]. Whereas the absolute power in the three frequency bands significantly overlap for tremor and non-tremor windows, a clear distinction between tremor and non-tremor windows is seen in the power distribution over the three frequency bands. It has to be noted that tremor classification was not based on power distributions or absolute power in the tremor band, but solely on the occurrence of a resonance peak in the PSD within the TF band. Because of the distinction in power distribution, the relative power was used in the scatterplots described below. Also in accordance with previous results, no significant differences were found in the power distribution patterns for the different DBS settings.

### Phase locking value

**Figure 3:**
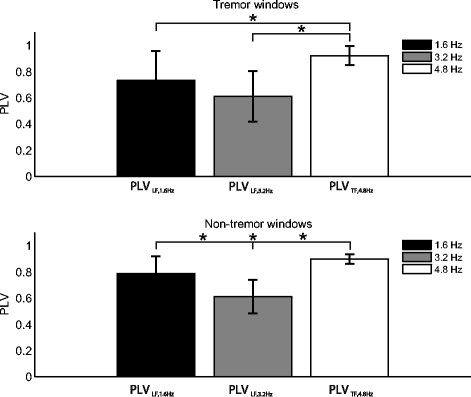
**The PLV for the three cueing frequencies averaged over all tremor (upper graph) and non-tremor (lower graph) windows.** The PLV expresses the synchronization of the movement related component of the angular velocity signal with the auditory cueing signal at 1.6 and 3.2 Hz (*PLV*_*LF,1.6Hz*_ and *PLV*_*LF,3.2Hz*_, respectively), and the tremor component of this signal with the cueing frequency of 4.8 Hz (*PLV*_*TF,4.8Hz*_). Statistically significant differences are indicated (*: p < 0.05); no statistically significant differences were found between the tremor and non-tremor windows.

Figure 3 plots the average PLV for the tremor windows (upper graph) and non-tremor windows (lower graph) for each of the three cueing frequencies. The left two bars of both graphs illustrate the degree of synchrony between the auditory cueing signal at 1.6 and 3.2 Hz and the LF component (<3.5 Hz, associated with voluntary movement) of the angular velocity signals recorded at hands and feet; the right bar shows the degree of synchrony between the highest cueing frequency (4.8 Hz) and the tremor component (3.5-7.5 Hz) of the recorded movement signals. Statistically significant differences are indicated (p < 0.05 and application of Bonferroni correction (n = 9)). No differences were found between tremor absence and presence. Only in case of tremor absence a significantly higher level of synchronization with the cueing signal of 1.6 Hz was found in comparison to the cueing frequency of 3.2 Hz. Interestingly, for both the tremor and non-tremor windows the level of synchronization of the tremor component with the cueing signal was higher than the movement component was with the lower cueing frequencies.

**Figure 4:**
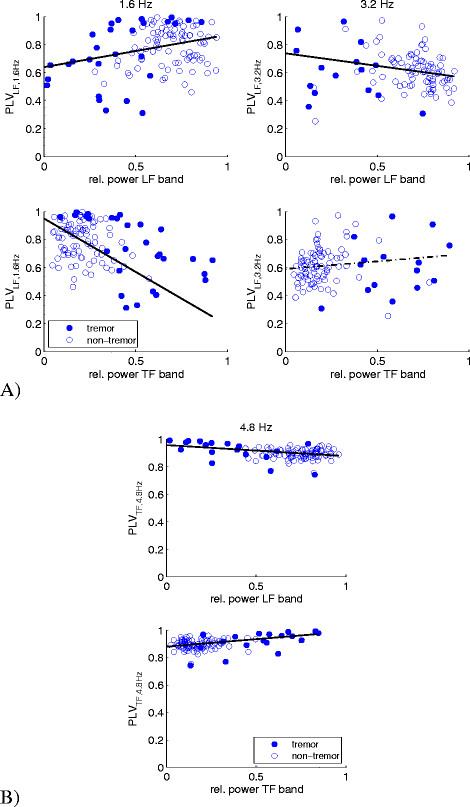
**The relationship between different PLVs and the relative power in different frequency bands of the angular velocity. (A)** Scatterplots of the *PLV*_*LF,1.6Hz*_ (left) and *PLV*_*LF,3.2Hz*_ (right) versus the relative power in the LF (top panels) and TF (bottom panels) bands of the angular velocity. Linear regression lines (solid line for p < 0.05 or a broken line for p > 0.05) are presented. **(B)** Scatterplots of the *PLV*_*TF,4.8Hz*_ versus the relative power in the LF (top panels) and TF (bottom panels) bands of the angular velocity recorded at hands and feet.

We tested whether trends could be observed from the power distribution in the LF and TF bands in relation to the PLV. Figure 4 plots the average *PLV*_*LF,1.6Hz*_ (Figure 4A - left)*, PLV*_*LF,3.2Hz*_ (Figure 4A - right)*,* and *PLV*_*TF,4.8Hz*_ (Figure 4B) as a function of the relative power in the LF (top panels) and TF bands (bottom panels) of the angular velocity. For increasing PLV values, the relative power in the LF band increases for a 1.6 Hz cue (p < 0.05) (Figure 4A - top left), but slightly decreases for a 3.2 Hz cue (p < 0.05) (Figure 4A - top right). The opposite is true for the relationship between the average *PLV*_*LF,1.6Hz*_ and *PLV*_*LF,3.2Hz*_ and the relative power in the TF band (Figure 4A – bottom). The average *PLV*_*TF,4.8Hz*_ shows a slight decrease with increasing relative power in the LF band (p < 0.05) (Figure 4B – top), and a slight increase in the TF band (p < 0.05) (Figure 4B – bottom). The level of entrainment of tremor to the cueing frequency was highest for those windows classified as tremor windows (closed markers).

### Tremor occurrence

**Figure 5:**
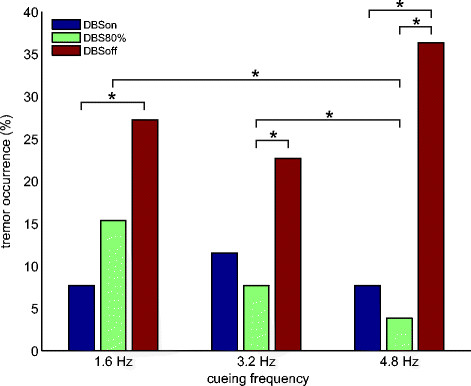
**The percentage of all extremities showing tremor during cued tapping movements at three cueing frequencies (1.6, 3.2, or 4.8 Hz) for three different DBS settings (DBSon, DBS 80%, DBSoff).** Statistically significant differences are indicated (*: p < 0.05).

Figure 5 shows the percentage of all extremities that showed tremor under the different cueing conditions and DBS settings. The Chi-square test was used to assess whether differences in the number of extremities showing tremor during the different testing conditions could have been produced by chance. Significant differences (p < 0.05) are indicated. The null hypothesis for each comparison states that the percentage of extremities exhibiting tremor is the same under both conditions and is equal to the percentage when both conditions are combined. DBS enhanced the effect of cueing, even for a metronome frequency of 4.8 Hz.

## Discussion

A clear distinction can be found between tremor and non-tremor windows in the power distribution of the signals recorded from the extremities during the performance of auditory cued movements. These two distinct patterns, in which there is an interchange of power between the LF and the TF bands, are analogous to those found for rest and action tremor during self-paced movements [32]. No statistically significant differences were found for the power distributions of hands and feet, and left and right side of the body.

While DBS may have affected the occurrence and severity of tremor, comparisons between the different DBS settings did not show differences in the synchronization of either movement or tremor component with the auditory cue. Therefore, the data for all DBS settings were combined to investigate the effect of auditory cueing on the synchronization of the movement and tremor component for different cueing frequencies, and the relation between the level of synchronization and the relative movement and tremor power (Figures 3 and 4). It may have been expected that auditory-cued movements could be better performed, especially when tremor was absent, resulting in a high PLV. Small differences were found by comparing the PLV of the three cueing frequencies. The synchronization of the tremor component with the auditory cue at the highest frequency (4.8 Hz) was most distinct (Figure 3). Furthermore, the presence or absence of tremor did not affect the PLV for the different frequencies. This is in line with the findings of Freeman et al., showing that the (in-)accuracy of the rhythmic finger tapping movements in the presence of auditory timing cues was not related to the presence or absence of tremor [42].

For the patients included in this study, the average tremor frequency of all extremities both at rest and during self-paced tapping movements was around 4.8 Hz [32]. The use of the three metronome frequencies did not significantly change the mean tremor frequency in case tremor windows were detected: 4.7 ± 1.0 Hz for 1.6 Hz cues; 4.8 ± 0.9 Hz for 3.2 Hz cues; 4.9 ± 1.0 Hz for 4.8 Hz cues. It may be expected that cueing may affect movement or tremor depending on the frequency. For a 1.6 Hz cue, the balance between the division of signal power into the LF band and TF band is in favour of the low frequency band (Figure 4A). Thus, the arm or foot movement becomes synchronized with the auditory cue. A contrasting trend is shown in case of a metronome frequency of 3.2 Hz. In this case the highest level of synchronization was reached when tremor was present, indicating that the tremor component, instead of the movement, may be weakly attracted by the cue (trend line for the TF band p > 0.05). For a metronome frequency of 4.8 Hz, only a small difference between tremor and non-tremor windows can be seen: the highest relative power in the TF band is associated with the highest level of synchronization when tremor is present (closed markers in Figure 4B). The high level of synchronization for even non-tremor windows may confirm previous conclusions that tremor may be present in PD patients without being clinically detectable [43, 44].

Although movement performance as expressed by the PLV under cueing conditions was not affected by the presence or absence of tremor, the combination of DBS and auditory cueing seemed to have a beneficial effect on action tremor (Figure 5). When electrical stimulation was on (DBSon), the number of extremities showing action tremor was significantly reduced compared to the stimulation off condition (DBSoff) for both the lowest (1.6 Hz) and the highest (4.8 Hz) cueing frequency (p < 0.05).

From previous studies on the use of external cues it may be proposed that providing neural input via pathways different from those through the basal ganglia may be sufficient to improve motor control in PD [19, 45]. The beneficial effect of cueing may result from the activation of compensatory processes via pathways such as the cerebellar loop [8, 9, 45]. In gait therapy, the cueing frequency of auditory (regular beeps), visual (flashing light), or somatosensory (pulsed vibrations) stimuli are often set such that it matches the rhythm of normal walking (~100 steps/min. = 1.67 Hz) [37]. However, current results suggest that the frequency of the cueing signal is not that critical. It may be, as previously suggested, that the rhythmic cueing acts to reset the pathological oscillatory activity in the basal ganglia [15], allowing the performance of voluntary movements. This may explain the observation that a cueing frequency close to the tremor frequency also has a beneficial effect. It may also confirm the involvement of the cerebellar circuits in the expression of tremor [19, 45, 46], and the proposed compensatory role of the observed tremor-related hyperactivity in the cerebellar loop in preventing tremor from spilling over into voluntary movement [8].

That the combination of DBS and cueing leads to a reduction of tremor occurrence may be related to the fact that the basal ganglia and cerebellar loops largely project onto the same cortical areas but are involved in different aspects of (motor) behaviour [47, 48]. Furthermore, Butson et al. have shown that STN DBS may also directly influence cerebellar projection areas: stimulation at electrode contacts that improve bradykinesia and rigidity generated volumes of activation that encompassed the fields of Forel (H2) and zona incerta (ZI), to which the cerebellum projects [49]. The cerebellar projection areas in ZI activated by STN DBS may affect functionally different cerebellar loops than those activated by cueing. Functional imaging techniques and EEG recording may provide a means to further test the involved mechanisms of DBS and cueing within the different cerebellar and cortical circuits, respectively.

It should be noted that the switching between different metronome frequencies during the test may have influenced the results. By randomizing the sequences for each test and patient, no differences were expected to exist between data collected using the three metronome frequencies. However, switching may have increased variations in the measurements, as it has been shown to be more difficult for Parkinson’s patients to switch from one cueing frequency to another [50]. Nevertheless, statistical significant differences were found.

Since we were only interested in tracking the pace of the movement set by the metronome and the occurrence of action tremor, it was not tested whether patients were accurately touching the indicated spots on the table or the floor with their hand or foot, respectively. It has been found that higher movement speed can only be realized at the expense of accuracy in PD patients [6]. However, the dots actually provided visual cues that may correct and regulate the scaling and amplitude generation problems that PD patients normally experience [37].

After changing the DBS setting patients were allowed to rest for about 5 minutes to adjust to the new setting. The expected effect of STN DBS on tremor has been found to occur within seconds of the onset of stimulation [51]. Vice versa, tremor also reoccurs within seconds when stimulation is switched off. However, bradykinesia and rigidity may show delayed responses to switching DBS on or off [52], which may have led to an underestimation of the effect of DBS in this study. This underestimation was expected to be similar across all stimulation settings, allowing the comparison of different stimulation settings [52]. Furthermore, patients were not forced to be off medication.

## Conclusions

Guided by external cues PD patients show an increased ability to perform repetitive movements, which has been proposed to result from activating cerebellar loops that effectively bypass the defective basal ganglia. Low cueing frequencies (<2 Hz) resulted in synchronization of the movement component while the tremor component of the recorded angular velocity signals synchronized with a cueing frequency that was near the tremor frequency. The presence or absence of tremor, however, did not affect the level of synchronization at any cueing frequency. The combined action of DBS and auditory cueing was found to lead to a significant reduction in the number of extremities showing action tremor, for all cueing frequencies tested.

## Authors’ original submitted files for images

Below are the links to the authors’ original submitted files for images. Authors’ original file for figure 1 Authors’ original file for figure 2 Authors’ original file for figure 3 Authors’ original file for figure 4 Authors’ original file for figure 5
