# Supplementary figures and images for: Effects of STN DBS and auditory cueing on the performance of sequential movements and the occurrence of action tremor in Parkinson’s disease
Source: J Neuroeng Rehabil. 2014 Sep 11;11:135. doi: 10.1186/1743-0003-11-135 (PMC4168195; doi:10.1186/1743-0003-11-135)

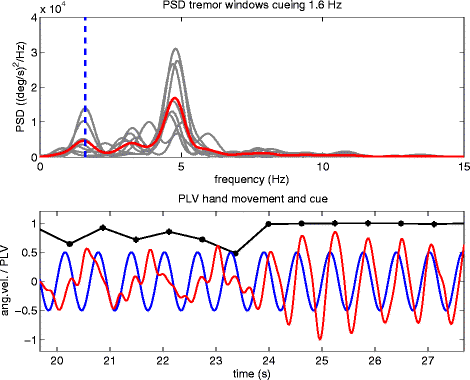

Supplement: Supplementary file 1 — Authors’ original file for figure 1 [file 12984_2014_655_MOESM1_ESM.gif]

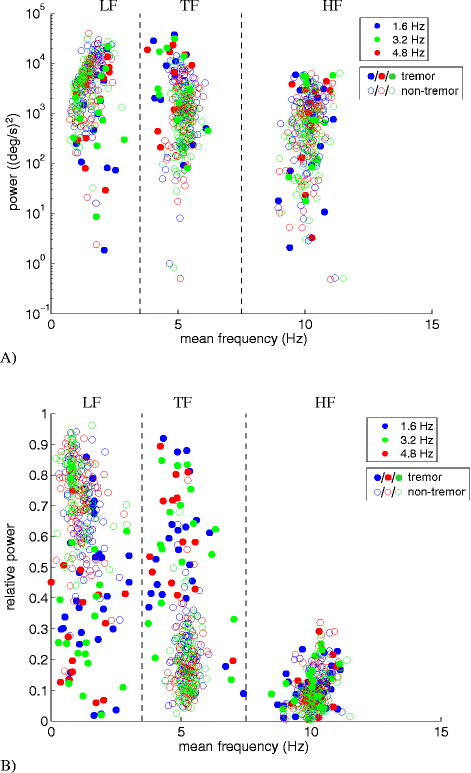

Supplement: Supplementary file 2 — Authors’ original file for figure 2 [file 12984_2014_655_MOESM2_ESM.gif]

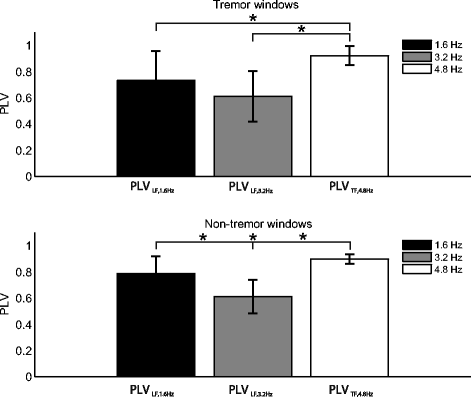

Supplement: Supplementary file 3 — Authors’ original file for figure 3 [file 12984_2014_655_MOESM3_ESM.gif]

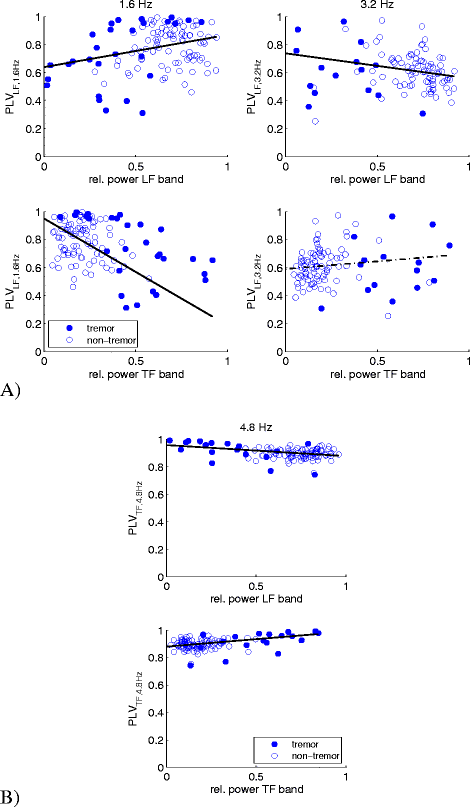

Supplement: Supplementary file 4 — Authors’ original file for figure 4 [file 12984_2014_655_MOESM4_ESM.gif]

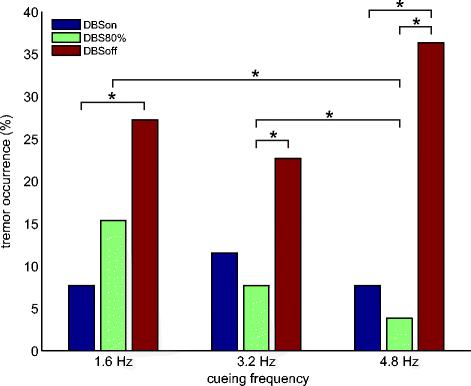

Supplement: Supplementary file 5 — Authors’ original file for figure 5 [file 12984_2014_655_MOESM5_ESM.gif]
